# Watchful waiting for some children with a mediastinal mass: the potential role for ^18^ F-fluorodeoxyglucose positron emission tomography: a case report and review of the literature

**DOI:** 10.1186/1471-2431-13-103

**Published:** 2013-07-10

**Authors:** Rosa Nguyen, Jamie L Coleman, Scott C Howard, Monika L Metzger

**Affiliations:** 1 Department of Pediatrics, University of Maryland, 22 S Greene St, Baltimore, MD 21210, USA; 2 Department of Radiological Sciences, St. Jude Children's Research Hospital, 262 Danny Thomas Place, Memphis, TN, USA; 3 Department of Oncology, St. Jude Children's Research Hospital, 262 Danny Thomas Place, Memphis, TN, USA; 4 University of Tennessee Health Sciences Center, 920 Madison Ave, Memphis, TN 38163, USA

**Keywords:** Mediastinal disease, Mediastinum, Positron-emission tomography, Thymus hyperplasia

## Abstract

**Background:**

Benign hyperplastic thymus is a rare but important differential diagnosis of anterior mediastinal lesions. Histological and radiological criteria are used to distinguish this benign condition from other malignant diseases but have their limitations, and biopsy of mediastinal masses can be risky. We report for the first time the diagnostic value of fluorodeoxyglucose ^18^ F positron emission tomography for patients with incidentally identified anterior mediastinal masses to avoid biopsy in some cases.

**Case presentation:**

A 2 year old girl presented with new onset of emesis and constipation leading to the incidental discovery of an anterior mediastinal mass on radiograph. Chest computed tomography revealed cystic components within the mass concerning for a malignancy. Biopsy of the lesion and bone marrow aspiration and biopsy were negative but there was concern that the mediastinal biopsy may have missed the malignant component of the lesion. Hence, a positron emission tomography scan was obtained that showed mild homogeneous fluorodeoxyglucose ^18^ F avidity within the mass similar to that of normal thymus. The diagnosis of benign hyperplastic thymus was made.

**Conclusion:**

The differential diagnosis of an incidentally found anterior mediastinal mass includes malignancy, but benign lesions such as benign hyperplastic thymus must also be considered, particularly when the complete blood count and biochemical profile are normal. Fluorodeoxyglucose ^18^ F positron emission tomography can help guide a clinician’s decision for further interventions and treatment.

## Background

Potential etiologies of anterior mediastinal masses in children include benign and malignant tumors whose incidences vary by patient age and symptoms at presentation. Benign hyperplastic thymus (BHT) is a rare condition that occurs mainly in infants and usually resolves spontaneously by 3 years of age, while it seldom occurs in older children and never in adults [1-3]. BHT is characterized by an increase in size of the thymus with normal histological architecture [1-5]. Hence, BHT must be distinguished from follicular hyperplasia in association with Graves’ disease, rebound thymus hyperplasia in cancer patients after chemotherapy, or thymoma and thymolipoma, which do not meet histological criteria for BHT and are mainly seen in adolescents and adults, respectively [1,6,7].

Diagnoses such as germ cell tumor, thyroid cancer, and lymphomas (Hodgkin and non-Hodgkin) must be ruled out during diagnostic workup. BHT spontaneously resolves over time, without specific treatment or surveillance; thus, prognosis and management differ from that of malignant etiologies [1,8]. Fine-needle aspiration cytology and imaging studies (e.g., chest radiograph, ultrasound, and computed tomography [CT]) are used to diagnose BHT in a minimally invasive manner but may result in inadequate biopsy specimens or inconclusive radiologic findings, requiring more invasive and potentially dangerous procedures [9-11]. In one study of 54 children and adults who underwent mediastinal biopsy, the procedure-related morbidity was 6%, and fatalities have been reported [12].

Here we describe a child whose diagnosis of BHT was based on CT-guided core needle biopsy and supported by conventional imaging and ^18^ F-fluorodeoxyglucose (FDG) positron emission tomography (PET) scan. PET can help differentiate BHT from other conditions associated with an anterior mediastinal mass.

## Case presentation

**Figure 1:**
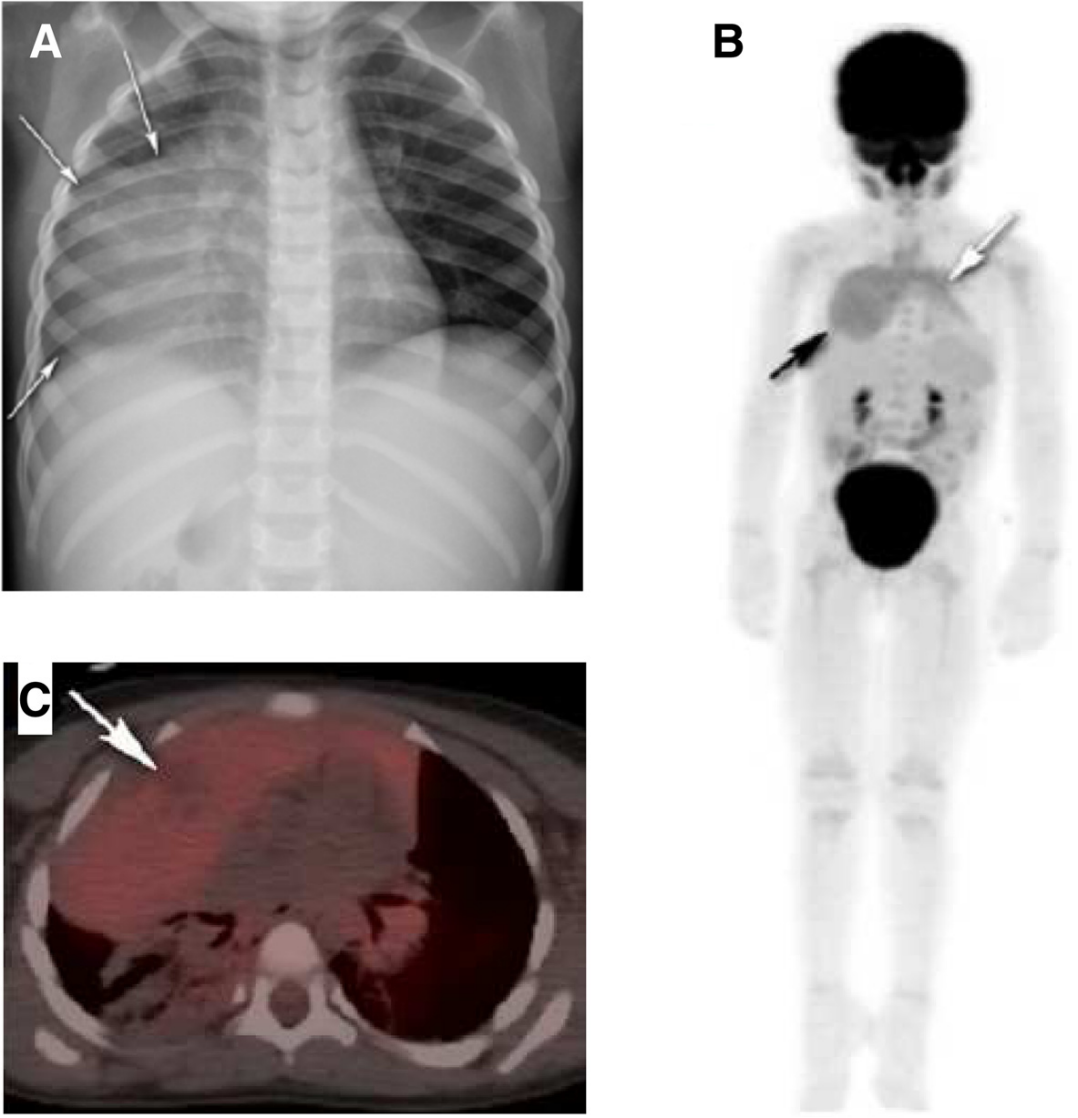
**Diagnostic imaging studies of the presented patient.** (**A**) Anteroposterior chest radiograph reveals a large predominately right-sided mass. (**B**) PET image shows that activity within the mass (black arrow) is indistinguishable from that in the normal thymus (white arrow). (**C**) Fused transaxial ^18^ F-FDG PET/CT shows only low-intensity, homogeneous ^18^ F-FDG avidity within the mass, with photopenia corresponding to the cystic/necrotic area (white arrow).

A 2-year-old girl with no significant past medical history was in her usual state of health when she developed non-bloody and non-bilious emesis associated with constipation. A review of other systems was negative. She did not take any medications and family history was non-contributory. In the emergency room, she appeared well and in no acute distress, with a normal physical examination. Weight, height, and vital signs were normal for age and gender. A chest/abdominal radiograph to evaluate her constipation showed no intra-abdominal pathology but demonstrated mediastinal widening (Figure 1A). A CT scan revealed a 10.3 cm × 6.6 cm × 6.3 cm heterogeneous, right-sided anterior mediastinal mass with a single hypodense area likely representing necrosis, and compression of bronchovascular structures. Laboratory tests showed no hematologic or metabolic derangements.

The patient underwent CT-guided core needle biopsy of the lesion, bilateral bone marrow aspirates and biopsies to rule out a malignancy. Hematoxylin and eosin staining and immunohistochemical studies of the mediastinal mass biopsy showed preservation of thymic architecture, with sheets of variably mature small- to intermediate-sized lymphoid cells and few Hassall's corpuscles, consistent with benign thymic tissue. Flow cytometry revealed T-lymphocytes with a full spectrum of orderly thymic maturation patterns, and bone marrow aspirates and biopsies showed normocellular marrow (80%) with trilineage hematopoiesis without evidence of leukemia or lymphoma.

However, the inhomogeneous area within the mass on CT **imaging** is not a typical finding of BHT and raised concern for a malignancy such as teratoma or lymphoma, which may **present with** such features (Table 1). PET-CT scan **of** the large anterior mediastinal mass **showedareas of** low-intensity, and diffuse, homogeneous FDG avidity, similar to **the** normal thymus (Figure 1B). Central low-attenuation areas by CT within the mass lacked FDG activity **and are** suggestive of necrosis (Figure 1C). The standardized uptake value of the lesions ranged from 2.3 to 2.5 MBq/kg, except for the cystic area, which had values less than 2 MBq/kg.

**Table 1:** Differential diagnosis of mediastinal masses in children

**Differential diagnosis**	**Diagnostic imaging modality**			**Comments**
	**Computed tomography**	**Magnetic resonance**	**PET scan**	
**Normal thymus**	Quadrilateral with convex or straight margins in infants, triangular with concave or straight margins in older children	Bilobed, convex at birth, straight during puberty, concave in old age, greater generalized T1-weighted and fast spin-echo T2-weighted hyperintensity and diminishing intermediate T1- and T2-signal soft tissue with fatty involution	Homogeneous low-intensity uptake	Age-dependent change in appearance
**Benign Etiologies**				
**Benign thymic hyperplasia**	Symmetrically enlarged, typically homogeneous	Enlarged, thymus characteristics similar to normal thymus (see above)	Typically homogeneous low-intensity uptake	Idiopathic
**Thymic follicular hyperplasia**	Symmetrically enlarged, normal sized in 25%-50%	Enlarged, thymus characteristics similar to normal thymus (see above)	Homogeneous uptake.	Chronic inflammatory states, autoimmune conditions, myasthenia gravis (65%-75%)
**Rebound hyperplasia**	Symmetrically enlarged, normal sized in 25%-50%	Enlarged, thymus characteristics similar to normal thymus (see above)	Increased homogeneous uptake	After chemotherapy
**Thymolipoma**	Pericardial fatty mass with fibrous septa	Hyperintense T1-signal resembling subcutaneous fat and area with intermediate intensity soft of tissue attenuation	Resembling uptake in fatty tissue and normal thymus	Mainly in adolescents and young adults
**Malignant Etiologies**				
**Lymphoma**	Homogeneous or heterogeneous, nodular, hemorrhage, necrosis, cystic components	Homogeneous low-signal on T1-weighted images, high-signal or intermixed areas (low and high) intensity on T2-weighted images	Heterogeneous intense uptake	Peak incidence in adolescence
**Teratoma**	Well-circumscribed, displacing mass, calcification (80%), fat-fluid levels, cystic, heterogeneous changes in lung parenchyma, pleura, or pericardium (tumor rupture)	Hyperintense fat on T1-weighted images within fluid of low signal intensity (cystic changes), hyperintense mass on T2-weighted images	Heterogeneously avid	Tissue from germ-cell layers
**Seminoma**	Large and lobular, homogenous	High-intensity mass with with septal structures in T2-weighted images	Heterogeneously avid	Most common primary solid tumor of the mediastinum
**Non-seminomatous tumor**	Large, lobulated, heterogeneous masses with large (>50%) areas of low attenuation, hemorrhage, necrosis	Internal heterogeneous intensities with areas of high signal intensity reflecting degenerative cystic changes on T2-weighted images.	Heterogeneously avid	Highly aggressive
**Thyroid carcinoma**	Well-defined, smooth or lobuated, tracheal deviation, contrast-enhancing, calcifications	Most tumors are hyperintense of markedly hyperintense on T2-weighted images	Heterogeneously avid	Ectopic thyroid

The diagnosis of BHT was made based on histology and lack of FDG avidity on PET scan. The patient continues routine follow up almost a year after original diagnosis with stable mediastinal mass and no further problems or complaints.

## Discussion

As the scope of diagnostic imaging broadens, there is greater likelihood of incidental detection of anterior mediastinal masses. Lymphomas and germ cell tumors are the most common malignant tumors of the anterior mediastinum in children; whereas, thymomas seldom occur in this age group [13]. Thymolipomas may occur in young adolescents and adults and occasionally children. Although their radiographic appearance may resemble BHT, thymolipomas present on MRI as mainly fatty masses with inhomogeneous areas that represent thymus tissue [14,15]. BHT is a rare but important benign condition and must be differentiated from malignant and other benign tumors.

To date, 53 patients with BHT have been reported (Table 2); 34 (64%) of them had respiratory symptoms, and imaging studies revealed a mediastinal mass. In the remaining 19 (36%) patients, the mediastinal lesion was an incidental finding. In either scenario, further diagnostic workup was warranted to rule out a malignancy; however, the implication of malignancy together with imaging findings and parental fear may have led to use of invasive interventions for diagnostic confirmation. Indeed, 79% (15/19) of asymptomatic patients underwent open biopsy and only 26% (9/34) of symptomatic patients were observed clinically without biopsy. In symptomatic patients, open biopsy can be diagnostic and therapeutic, but less invasive procedures are preferred [16].

**Table 2:** Reported cases of benign thymic hyperplasia

**Reference**	Case no*	**Sex**	**Age**	**Size**	**Presenting symptoms**	**Diagnostic imaging**	**Pathology**	**Course**	**Comments/Follow up**
**Symptomatic cases**									
Oh [17]	1	F	15	15 × 10 × 2 cm, 102 g	Pulmonary infection	Fluoroscopy, angiography	Histology	Open resection	None
	2	F	14	8.4 × 2.8 × 1.4 cm, 20 g	Upper respiratory infection	Fluoroscopy	Histology	Open resection	None
Ruco [5]	-	M	5	950 g	Dyspnea	None	Histology	Open resection	None
O'Shea [18]	-	M	1	420 g	Dyspnea, lymphocytosis	CXR	FNA, histology	Steroids, open resection	5 months
Barcia [19]	2	M	4	47-92 g	Pulmonary infection	CXR	Histology	Open resection	1 month
	3	M	1	47-92 g	Pulmonary infection	CXR	Histology	Open resection	1 month
	11	F	9	47-92 g	Chest discomfort	CXR	Histology	Steroids, open resection	1.5 years
Rasore-Quintino [20]	-	M	4	800 g	Pulmonary infection	^99^Technetium scan	Histology	Open resection	None
Lack [6]	2	M	11	15.2 cm, 324 g	Mild dyspnea, URI	CXR	Histology	Open resection	9 years
Lamesch AJ [21]	-	M	6/12	230 g	Respiratory distress	CXR	Histology	Ventilation, steroids, open resection	6 years
Parker [11]	-	M	1 3/12	200 g	Pulmonary infection	CXR, US, fluoroscopy, CT	Histology	Open resection	None
Kobayashi [22]	1	M	1/12	-	Respiratory distress, lymphocytosis	CXR, CT	None	Observation, steroids	Intensive care unit admission, no follow-up
	2	M	2/12	-	Respiratory distress, lymphocytosis	CXR, CT	None	Observation, steroids	None
	3	M	4/12	-	Pulmonary infection, lymphocytosis	CXR, CT	None	Observation, steroids	None
	4	F	1/12	-	Respiratory distress, lymphocytosis	CXR, CT	None	Observation	None
Nezelof [23]	1	F	10	93 g	Cough	CXR	Histology	Open resection	None/uneventful follow up
Judd [24]	-	M	12	13 × 8 × 3.5 cm, 245 g	Wheezing, dysphagia	CXR	Basic laboratory tests, histology	Open resection	None
Ricci [2]	3	M	14	850 g	Dyspnea, altered LFTs, atelectasis	CXR, CT	ECG, LFTs, histology	Open resection	9 years
	4	M	5	950 g	Dyspnea	CXR, CT	ECG, EMG, LFTs, biopsy ×2, histology	Open partial resection	Wound infection, osteomyelitis, lung atelectasis/1 month
Linegar [8]	1	F	2/12	220 g	URI, lymphocytosis, splenomegaly	CXR, CT	Histology	Open resection	3 months
	2	M	3	18 × 10 × 6 cm, 855 g	Recurrent URI, lymphocytosis	CXR	Histology	Open resection	None
	3	M	6	1260 g	Wheezing, dyspnea, respiratory distress	CXR, CT	FNA, histology	N/A	None
	4	M	3	100 g	Recurrent URI	CXR	Histology	Open resection	None
Lee [25]	1	F	3/12	-	Persistent URI, lymphocytosis	CT	Open biopsy, histology	Observation	1 year
	2	M	11/12	500 g	URI, lymphocytosis, mediastinal shift	CT	Histology	Open resection	None
Bangerter [9]	8	M	1/12	5x6 cm	Acute airway obstruction	Imaging not further specified	U/S guided FNA, histology	Steroids	Death 10 months after diagnosis of unknown cause/8 months
Hoerl [10]	-	M	5/12	4.6 cm AP	Choking	CT	FNA, histology	Observation	1 year
Tareen [26]	1	M	3/12	-	Persisting URI	CT, CXR, US	None	Steroids, observation	6 months
	2	M	8/12	-	Recurrent URI, dyspnea, tachypnea	CXR, CT	FNA, histology	Open resection	6 months
Sosothikul [27]	-	M	4	-	Dyspnea, wheeze	CXR, CT	BMA, histology	Observation	Involution/1 month
Gow [28]	-	F	6/12	N/A	Respiratory symptoms	Imaging not further specified	Flow cytometry, histology	Open resection	1 year
Piednoir [29]	-	M	3/12	-	Anesthesia related respiratory distress, incidental finding	CT	None	Observation	Involution/2 years
Szarf [30]	-	M	2	830 g	Fever, dry cough and dyspnea	CXR, CT	Alpha-FP, beta-HCG, FNA, histology	Steroids, open resection	Reoccurrence of symptoms
Tan [31]	-	F	9/12	17.5 × 11 × 5	Upper respiratory infection	CXR, MRI	FNA, histology	Steroids, open resection	None
**Asymptomatic cases**									
Oh [17]	3	F	10	80 g	Incidental finding	Fluoroscopy	Basic laboratory tests, histology	Open resection	None
Katz [32]	-	M	7/12	9 × 8 × 6 cm, 224 g	Incidental finding, lymphocytosis	CT, upper GI, IV pyelography	Immunologic studies, BMA, histology	Open resection	Hypogamma-globulinemia/4 years
Barcia [19]	1	M	4	47-92 g	Incidental finding	CXR	Histology	Open resection	1 year
	5	F	11	47-92 g	Incidental finding	CXR	Histology	Steroids, open resection	3 months
	6	F	3	47-92 g	Incidental finding	CXR	Histology	Open resection	3 months
	7	M	4	47-92 g	Incidental finding	CXR	Histology	Open resection	1 year
	8	M	4	47-92 g	Incidental finding	CXR	Histology	Open resection	2 years
	9	F	7	47-92 g	Incidental finding	CXR	Histology	Steroids, open resection	1.5 years
	10	M	13	47-92 g	Incidental finding	CXR	Histology	Open resection	4 months
Lee [33]	-	F	2	19 × 12 × 4.5 cm	Incidental finding	CXR	Histology	Oral steroids, open resection	None
Lack [6]	1	M	14	490 g	Incidental finding, lymphocytosis	CXR	Histology	Open resection	17 years
Nezelof [23]	2	F	5	N/A	Incidental finding	CXR, mediastinoscopy	Basic laboratory tests, biopsy, histology	Observation	None/uneventful follow up
	3	F	11	N/A	Incidental finding	CXR, mediastinoscopy	Biopsy, histology	Observation	None
Arliss [34]	-	M	15	17 × 16 × 6 cm, 680 g	Incidental finding, lymphocytosis	CXR, CT	Histology	Open resection	1 4/12 years
Ricci [2]	1	M	16	13 cm, 250 g	Incidental finding	CXR, CT	ECG, EMG, LFTs, histology	Open resection	12 years
	2	M	12	7.5 cm, 120 g	Incidental finding	CXR, CT	ECG, EMG, LFTs, histology	Open resection	7 years
Rice [3]	-	M	10	482 g	Incidental finding	MRI	BMA, histology	Open resection	None
Bangerter [9]	1	F	5	3 × 5	N/A	Imaging not further specified	U/S guided FNA, histology	N/A	9 years
	6	F	8	1.5 × 1	N/A	Imaging not further specified	U/S guided FNA, immunopheno-typing, histology	N/A	8 months
Current case	-	F	2		Incidental finding	CRX, CT, PET	Core needle biopsy	Observation	

* If number of patients with benign thymus hyperplasia and patients with other conditions is provided in report.

*CT* Computed tomography, *CXR* Chest radiograph, *ECG* Electrocardiogram, *EMG* Electromyogram, *FNA* Fine needle aspiration, *LFTs* Liver function tests, *MRI* Magnetic resonance imaging, *N/A* Not applicable, *PET* Positron emission tomography, *URI* Upper respiratory tract infection, *U/S* Ultrasound.

**Figure 2:**
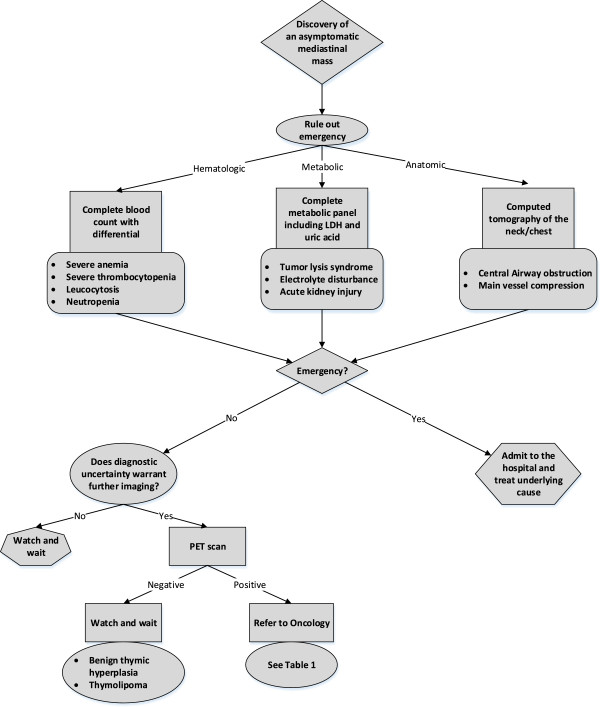
Flow chart for the workup of a mediastinal mass in an otherwise asymptomatic child.

The primary goal while assessing a mediastinal mass is to rule out oncologic emergencies (anatomic, metabolic, or hematologic) that require immediate medical attention. Workup includes patient’s history, physical examination, routine laboratory tests, and anatomic imaging (Figure 2). After excluding oncologic emergencies, further tests are needed to diagnose the mediastinal lesion.

Physiologic imaging, most often PET-CT, is recommended in the diagnostic process when uncertainty about the malignant versus benign nature of the mass persists (Figure 2) [35].

The pattern and intensity of uptake within such lesions on PET and morphologic appearance on CT can help differentiate benign from malignant etiologies [36] (also see Table 1). To accurately interpret BHT in PET studies, FDG uptake patterns in the normal thymus and pathologic entities involving the thymus need to be known [37]. Normal thymic tissue and benign conditions such as BHT after chemotherapy (so called “thymic rebound”), comparable to our patient, demonstrate diffuse, low-intensity, homogeneous FDG avidity (Table 1) [38,39].

Malignant conditions show intense FDG avidity that is usually heterogeneous in distribution [40]. The appearance of our patient’s lesion was similar to the normal thymus without focality, suggesting its benign nature (Figure 1B), [41] despite the cystic and necrotic areas within the mass [36,42]. The morphology and uptake pattern on ^18^ F-FDG PET are more meaningful than the SUV. There is an overlap in SUV range between normal thymus and other malignant anterior mediastinal tumor entities [43]. Although a very high SUV may be indicative of malignancy, an average SUV does not exclude malignancy [36,43].

## Conclusion

In conclusion, for incidentally found anterior mediastinal masses in otherwise healthy children, we recommend that clinicians expeditiously rule out oncologic emergencies then perform a diagnostic workup. PET scans can help differentiate BHT from other more serious conditions and may spare patients invasive diagnostic procedures.

Further studies including large pediatric series are needed to evaluate the importance of ^18^ F-fluorodeoxyglucose positron emission tomography in patients with suspected benign hyperplastic thymus.

### Consent

Written informed consent was obtained from the patient’s mother for publication of this Case report and any accompanying images. A copy of the written consent is available for review by the Editor of this journal.

## Abbreviations

BHT: Benign hyperplastic thymus; BMA: Bone marrow aspiration; CT: Computed tomography; FDG: ^18^ F- fluorodeoxyglucose; PET: Positron emission tomography; SUV: Standardized uptake value.

## Competing interests

The authors declare that they have no competing interests.

## Authors’ contributions

All authors have participated in study design, interpretation, and writing of the report. RN did the collection of the data, review of literature, and drafted the first version of the manuscript. JLC provided the figures and reviewed the manuscript. SCH reviewed the manuscript. MLM primarily participated in study design and coordination and helped to draft the manuscript. All authors read and approved the final manuscript.

## Pre-publication history

The pre-publication history for this paper can be accessed here:

http://www.biomedcentral.com/1471-2431/13/103/prepub
